# Excited-State Dynamics Simulations of a Light-Driven Molecular Motor in Solution

**DOI:** 10.1021/acs.jpca.3c05841

**Published:** 2023-11-02

**Authors:** Jin Wen, Sebastian Mai, Leticia González

**Affiliations:** † State Key Laboratory for Modification of Chemical Fibers and Polymer Materials, College of Materials Science and Engineering, Donghua University, Shanghai 201620, China; ‡ Institute of Theoretical Chemistry, Faculty of Chemistry, University of Vienna, Währinger Str. 17, Vienna 1090, Austria

## Abstract

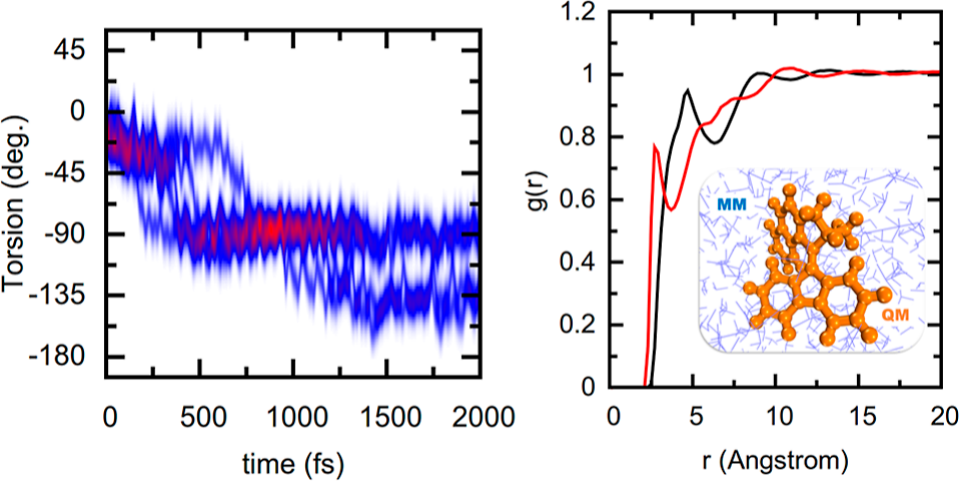

Molecular motors, where light can be transformed into motion, are promising in the design of nanomechanical devices. For applications, however, finding relationships between molecular motion and the environment is important. Here, we report the study of excited-state dynamics of an overcrowded alkene in solution using a hybrid quantum mechanics/molecular mechanics (QM/MM) approach combined with excited-state molecular dynamics simulations. Using QM/MM surface-hopping trajectories, we calculated time-resolved emission and transient absorption spectra. These show the rise of a short-lived Franck–Condon state, followed by the formation of a dark state in the first 150 fs before the molecular motor relaxes to the ground state in about 1 ps. From the analysis of radial distribution functions, we infer that the orientation of the solvent with respect to the molecular motor in the electronic excited state is similar to that in the ground state during the photoisomerization.

## 1 Introduction

Artificial molecular machines are assemblies that allow the conversion of chemical or light energy into mechanical work that accomplishes a useful task.^1,2^ Among them, synthetic molecular rotatory motors have attracted a lot of interest for their potential applications in biomolecular sensors, electronic devices, or photoswitchable catalysts.^3,4^ In a motor, mechanical work is achieved by a large amplitude motion, e.g., a full cycle of unidirectional rotation, after which the system returns to its original position.^1^ The chiroptical overcrowded alkenes developed by Feringa and co-workers^5−8^ are a good example of rotatory molecular motors. There, chirality and the asymmetry of the potential energy surfaces ensure unidirectional rotation around a double bond, exploiting sequential four-step cycles that include *cis*/*trans* photoisomerization, followed by a thermally controlled helix inversion that effectively hinders reverse rotation.

**Figure 1:**
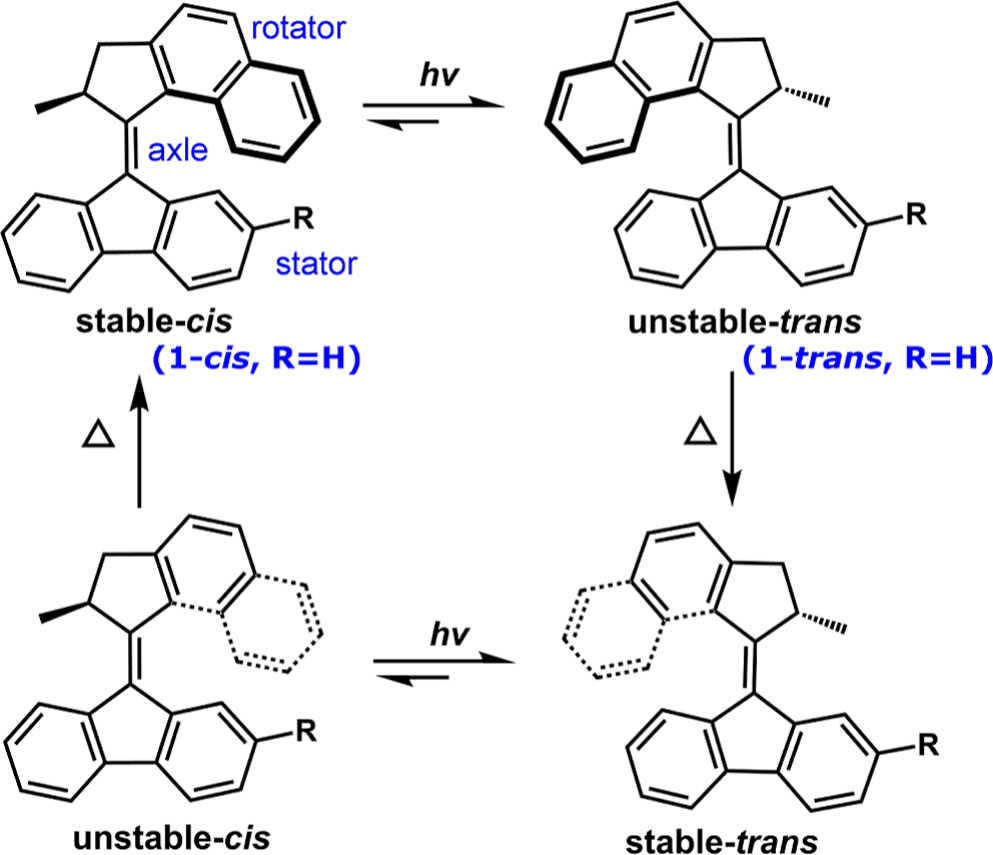
Schematic unidirectional rotation process of an overcrowded alkene-based molecular motor.^9^

While the first generation of motors based on overcrowded alkene motors had two identical halves connected by the carbon double bond,^5^ later developments included different rotator and stator parts—as in the example of Figure 1—thereby achieving significant rotary acceleration.^9^ Efficiency is limited by both the quantum yield of the photoisomerization and the reaction barrier associated with the thermal helix inversion step. Factors related to both thermal and photo reactions, including experimental parameters and molecular properties, were kinetically analyzed in solution.^10^ This analysis revealed that the rotational speed is not significantly influenced by the quantum yield. Conversely, increasing the size of the substituent at the stereogenic center in second-generation molecular motors offers a practical means to accelerate rotation by reducing the reaction barrier of helix inversion.^11^ Newer generations of molecular motors aim at increasing their rotational frequency up to the MHz scale.^9,12−16^ In this endeavor, precise spatiotemporal control is best achieved if the working rotary process is well understood and its collective behavior in solution or on attached surfaces^17,18^ can be predicted at the molecular level. Alternatively, recent theoretical predictions suggest that electricity could facilitate unidirectional rotation in these molecular motors, as anionization reduces the reaction barrier of the isomerization reaction.^19^

The light-activated step of a molecular motor is best monitored by time-resolved spectroscopy.^20^ Solution plays a role^21^ as the viscosity and polarity of the medium affect the photochemical quantum yield and the thermal reactions.^22,23^ Photodynamics of molecular motors have been investigated in both polar and nonpolar solvents^3,4,9,20,24,25^, indicating that the design of molecular motors, in particular, conditions of polarity and viscosity can be obtained from computational modeling that predicts the effect of a particular solvent on the rotary cycle.

The electronic structure and dynamics of the “dark state” have been a topic of debate in Feringa’s second-generation motors since its initial observation in ultrafast spectroscopy as it holds a crucial role in illustrating the potential energy surface and the photodynamics process.^20^ Utilizing high time-resolution UV-pump/IR-probe spectroscopy, researchers identified the dark state as the second excited state, closely positioned in energy to the first excited state.^26^ Subsequent theoretical dynamics simulations challenged this identification, suggesting that the S_2_ state was too energetically high for the photoisomerization process.^27^ Further investigations using time-resolved spectroscopy with a 50 fs resolution indicated that the initial 100 fs relaxation period was independent of solvent viscosity.^24^ Nevertheless, it was discovered that the decay of the dark state to the ground state within 1.6 ps is affected by both the substituent groups of the molecular motor and the solvent viscosity.^25^ It would be intriguing to simulate the photoisomerization of these molecular motors while explicitly incorporating the solvent to observe the combined effects of both the solvent and molecular properties.

Electronic structure calculations have been used previously to investigate the isomerization dynamics of the light-induced step of the second-generation motors.^28−30^ Wave packet dynamics based on a single effective coordinate with two electronic states in a dissipative environment were used as a model rotary motor that includes both the isomerization and thermal steps.^31^ Different theoretical studies have contributed to designing molecular motors with faster rotation and higher quantum efficiency.^32−34^ Yet, most studies connecting ultrafast dynamics in molecular motors with time-resolved dynamics simulations are done in the gas phase,^28,35,36^ with exceptions.^37,38^ The inclusion of a solution environment surrounding a chromophore is possible using a hybrid quantum mechanics/molecular mechanics (QM/MM) approach.^39^ Therefore, it is important to consider the influence of the solvent in a dynamic form in the discussion of the performance of the rotatory process in molecular motors. Thus, in this paper, we set out to investigate the coupled solvent–solute dynamics after the excitation of a molecular motor using QM/MM surface hopping simulations. As a molecular model, we chose compound **1**-*cis* (depicted in Figure 1). Experimental investigations on this molecule were performed in various solvents.^20,25^ As one focus of our work is coupled solvent–solute dynamics, we deemed good solubility an important criterion and thus selected dimethyl sulfoxide (DMSO) for our study, given its generally good solubility for molecular motors.^40^

## 2 Computational Methods

**Figure 2:**
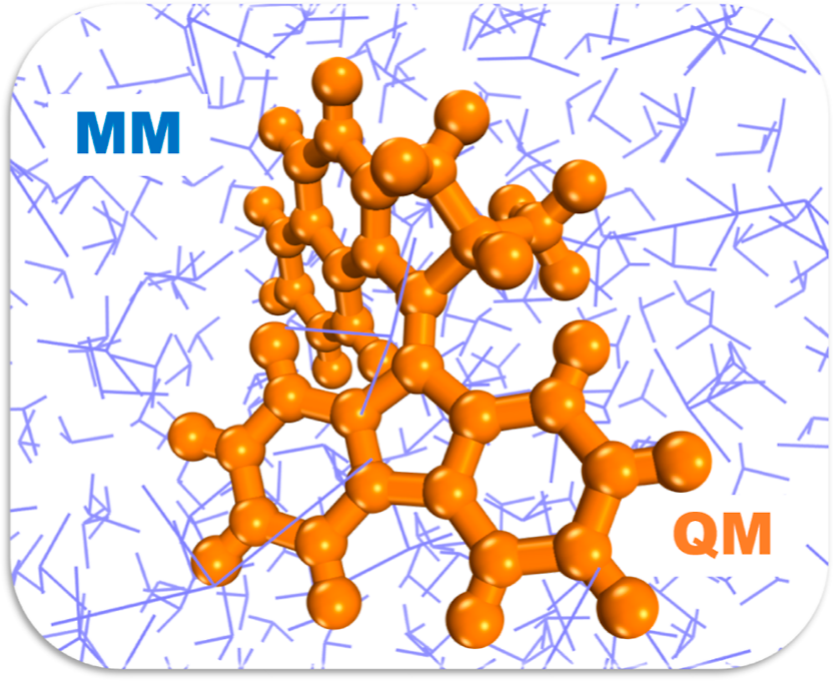
Schematic QM/MM model: the molecular motor in the QM region (orange) and the DMSO solvent in the MM region (blue).

In order to investigate the excited-state dynamics of **1**-*cis*, we employed excited-state dynamics simulations based on surface hopping trajectories,^41^ as implemented in the surface hopping including arbitrary couplings (SHARC)^42,43^ software. The inclusion of explicit solvents is achieved using QM/MM with electrostatic embedding.^44^ The QM part consists of the molecular motor chromophore (Figure 2), while the solvent molecules are treated classically by a force field in the MM part.

The methodological protocol has two steps. In the first step, classical molecular dynamics (MD) simulations in the electronic ground state were performed to generate an ensemble of initial conditions that could be used for subsequent excited-state QM/MM calculations. In the second step, surface-hopping trajectories in the electronic excited state were initialized and propagated in the QM/MM framework to ultimately deliver an estimate of the relaxation time, time-resolved emission and transition absorption spectra, and solvent–solute distributions. The spectra were compared with available experimental time-resolved signals.^20,25^ In the following, the computational details involved in these steps are specified.

### 2.1 Classical MD Simulations

Classical MD simulations for **1**-*cis* in DMSO were employed to generate initial conditions from where SHARC trajectories would propagate subsequently within the QM/MM framework. In the MD simulations, the general AMBER force field (GAFF)^45^ and charges derived from a restrained electrostatic potential (RESP) fit^46^ at the HF/6-31G(d) level were used for the molecular motor, which was solvated by 1434 DMSO molecules in a 12 Å truncated octahedron box. Since the parameters of the solvent DMSO were not included in the GAFF, the solvent box was parametrized according to ref (47). After minimization of the starting structure, a trajectory was propagated in the *NVT* ensemble for 100 ps to heat the system to 300 K, with a time step of 0.5 fs and without the SHAKE algorithm. The solvated box was equilibrated to 1 bar and 300 K for 1 ns in the *NPT* ensemble, followed by 10 ns production using the same conditions. A total of 200 snapshots (coordinates plus velocities) were extracted from these 10 ns production simulations at intervals of 50 ps to generate 200 initial conditions for the excited-state dynamics simulations, as described in the next subsection. The classical MD simulations were performed in the AMBER18 suite^48^ using periodic boundary conditions.

In order to quantify solute–solvent interactions, radial distribution functions (RDFs) were evaluated for atom pairs along the trajectories. The RDFs were calculated as
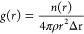
1where *n*(*r*) is the average number of solvent molecules in a spherical shell between radii of *r* and *r* + Δ*r*, and ρ is the particle density. The *g*(*r*) of the distances between C(motor)–S(DMSO) (noted as *g*_C–S_), C(motor)–O(DMSO) (*g*_C–O_), H(motor)–S(DMSO) (*g*_H–S_), and H(motor)–O(DMSO) (*g*_H–O_) were obtained with AmberTools^48^ from the 10 ns dynamics, with a bin size of Δ*r* = 0.3 Å. Using an in-house code, we also computed 2D correlated RDFs to reveal the correlation between the *g*_C–S_ and *g*_C–O_ RDFs. Using the combination of these one and two-dimensional RDFs, the orientation of solvent with respect to the chromophore can be estimated.

### 2.2 Electronic Structure Calculations

In the present work, we focus on the description of the initial dynamics of the molecule in the excited state, the corresponding time-dependent electronic spectra (emission and transient absorption), and the coupled dynamics of the solvent. Optimally, the level of theory should also be able to describe the passage through the twisted S_1_/S_0_ conical intersection (CI)^49−51^ and the subsequent isomerization dynamics. However, the correct description of this CI^52^ and dynamics requires a multiconfigurational or multireference level of theory. Single reference methods such as the second-order algebraic-diagrammatic construction [ADC(2)] scheme for the polarization operator^53^ or time-dependent density functional theory (TD-DFT)^54^ are computationally more efficient and can describe the populated excited state—and also higher excited states needed for simulating a transient absorption spectrum—accurately, as long as the dynamics does not approach the S_1_/S_0_ CI. In this work, we employ the ADC(2) method with the cc-pVDZ basis set^55^ for the QM part of the QM/MM calculations, for reasons of efficiency. This includes single-point calculations for the 200 snapshots taken from the classical MD simulation (including S_0_, S_1_, and S_2_ to generate an absorption spectrum and select initial conditions), the excited-state SHARC trajectories (including S_0_ and S_1_), and the single-point calculations needed for the transient absorption spectrum calculations (including 20 excited singlet states). These calculations were performed with TURBOMOLE 7.1.^56^ We also optimized a small number of structures in the ground state for further discussion, which were optimized at the B3LYP-D3/cc-pVDZ level and confirmed by subsequent frequency calculations. These computations were performed with the Gaussian 16 software package (Version A.03).^57^ The optimized coordinates are given in Tables S1–S3 in the Supporting Information.

### 2.3 QM/MM Excited-State Dynamics

The QM/MM-SHARC dynamics were carried out using SHARC 2.1.^42,43,58^ The QM region (the rotor) was computed with ADC(2) as described above, whereas the MM region was described with TINKER.^59^

According to the experimental excitation wavelength^5^ of 400 nm (3.10 eV) and the ADC(2)-simulated absorption spectrum of **1**-*cis* (presented below), only two singlet states (S_0_ and S_1_) were needed in the excited-state dynamics simulations after excitation to the first absorption band. Thus, we performed single-point calculations of the S_0_ and S_1_ for all 200 MD snapshots and found that in 133 snapshots, the S_1_ was the bright state. Out of these 133 snapshots, we stochastically selected 23 to run excited-state dynamics for up to 1 ps with a nuclear time step of 0.5 fs. Other snapshots were not excited, although stochastic selection ensured that the smaller set of 23 snapshots was consistent with the phase-space distribution of the 133 snapshots. We note that the small number of trajectories is due to the high computational cost of the ADC(2)/MM electronic structure calculations (about 10,000 CPUh per trajectory).

As ADC(2) does not correctly describe the nonadiabatic couplings between S_0_ and S_1_,^52^ we simply run the surface hopping simulations until the S_1_–S_0_ energy gap is below 0.1 eV.^60^ This provides a lower limit for each trajectory’s lifetime in the excited state and thus the overall S_1_ decay time. The trajectories were not continued in the ground state.

The time-resolved emission spectrum was computed from the S_1_–S_0_ energy gaps and oscillator strengths obtained from 23 trajectories. The data were convoluted with a two-dimensional Gaussian with a full width at half-maximum (fwhm) of 2.0 eV × 50 fs. For the transient absorption spectra, geometries were extracted every 25 fs from the 23 trajectories. At these geometries, the 20 lowest excited states were calculated with ADC(2)/cc-pVDZ and the S_1_–S_*n*_ energy gaps, and oscillator strengths were extracted and convoluted with a 0.3 eV Gaussian. RDFs from the surface hopping trajectories were averaged over the last 100 fs of the trajectories.

## 3 Results and Discussion

### 3.1 Ground-State Dynamics in Solution and the Ground-State Spectrum

**Figure 3:**
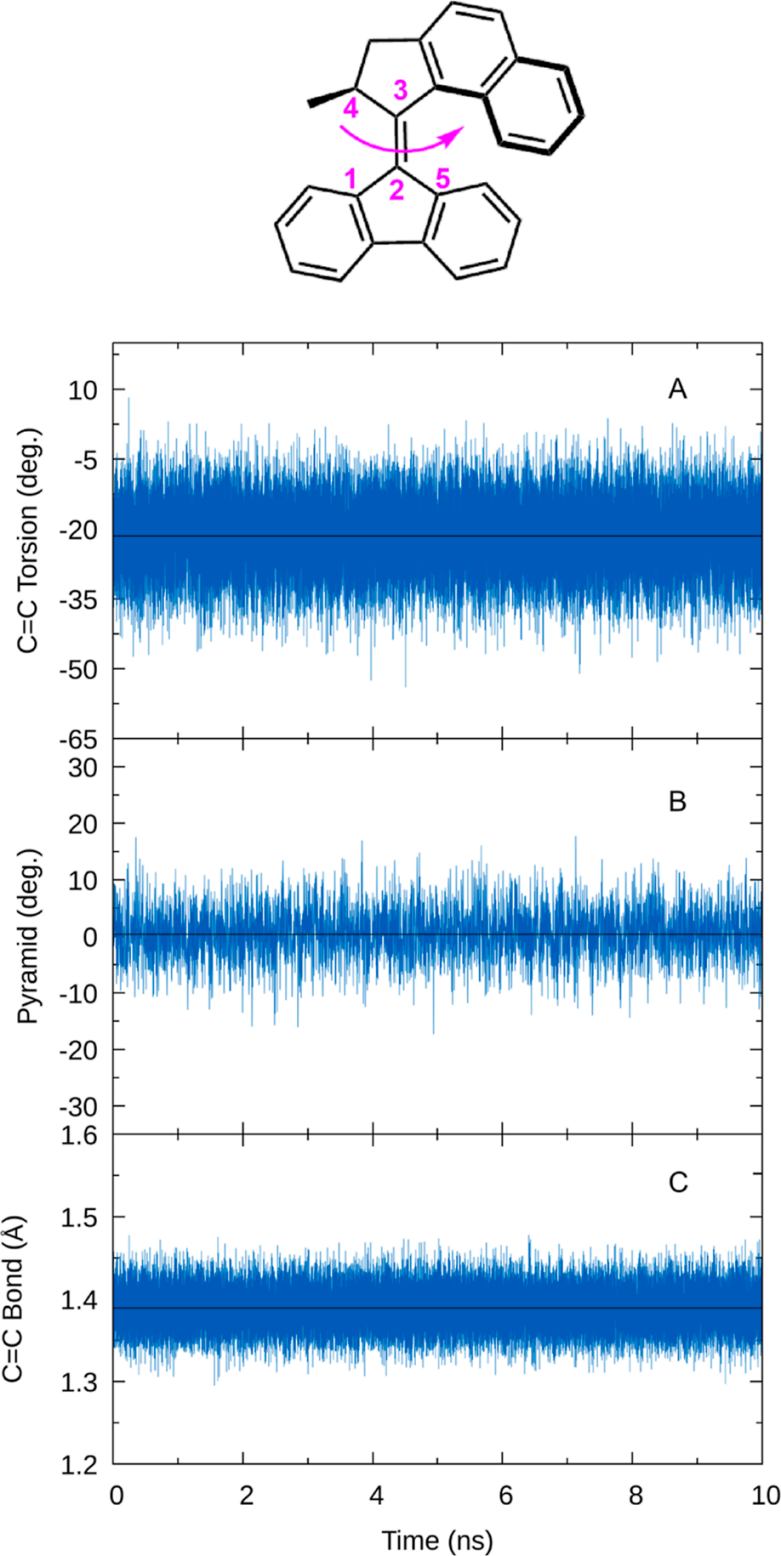
Ground state evolution of (a) C=C torsion, defined as the torsion between atoms 1–4, (b) pyramidalization, defined as the pyramidalization angle between atoms 1, 3, 5, and 2, and (c) C=C bond length between atoms 2 and 3 in **1**-*cis* during 10 ns. Average values are depicted as black solid lines.

From the classical MD simulations, we display in Figure 3 the time evolution of the most important geometry parameters of the initial motor in DMSO, which is in the stable-*cis* configuration (Figure 1). These include the C=C torsion angle, the pyramidalization angle, and the C=C bond length. As can be seen, the motor retains its original structure, and the stator retains its planar conformation in the presence of the highly viscous and polar solvent DMSO during 10 ns of dynamics.

**Figure 4:**
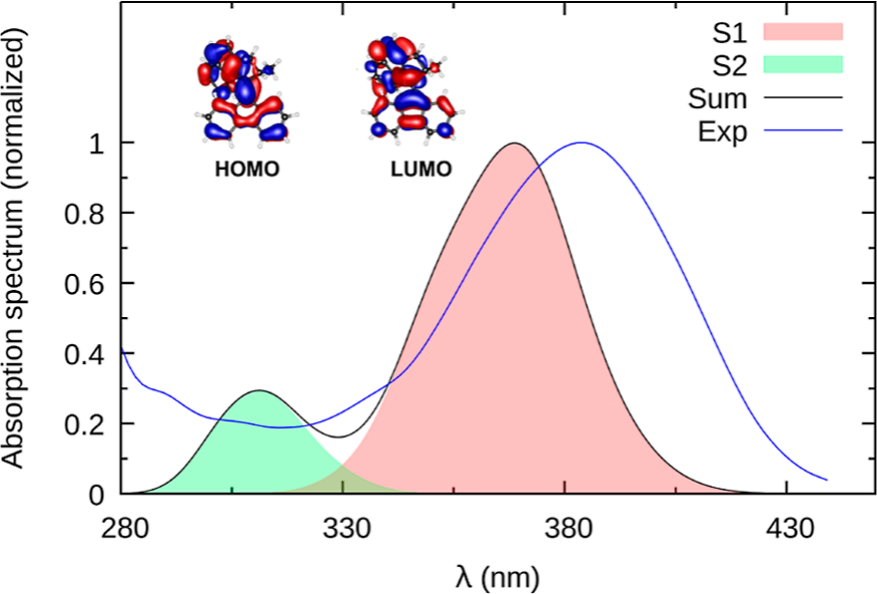
Simulated absorption of **1**-*cis* was based on 200 MD snapshots and computed with ADC(2)/MM for two excited states. Obtained by Gaussian convolution (FWHM of 0.2 eV). The first peak in the experimental^9^ spectrum is indicated by the blue line. The inset shows the frontier orbitals describing the bright S_0_–S_1_ transition.

Based on the 200 initial conditions obtained from the classical MD simulations, we simulated an ADC(2)/MM ground state absorption spectrum including the first two excited states. As shown in Figure 4, the spectrum peaks at 3.35 eV (about 370 nm), which agrees reasonably with the experimental^9^ absorption band in hexane indicated by the blue line. The electronic character of this band corresponds to a HOMO–LUMO excitation from delocalized π and π* orbitals with a large contribution at the central C=C bond (see the inset in Figure 4). We note that the simulated spectrum nicely shows that the S_1_ state is sufficient to describe the entire first absorption band, whereas S_2_ describes the onset of the second band. Consequently, only S_1_ is required to describe the dynamics after excitation to the first band.

### 3.2 Photoisomerization Dynamics in Solution

**Figure 5:**
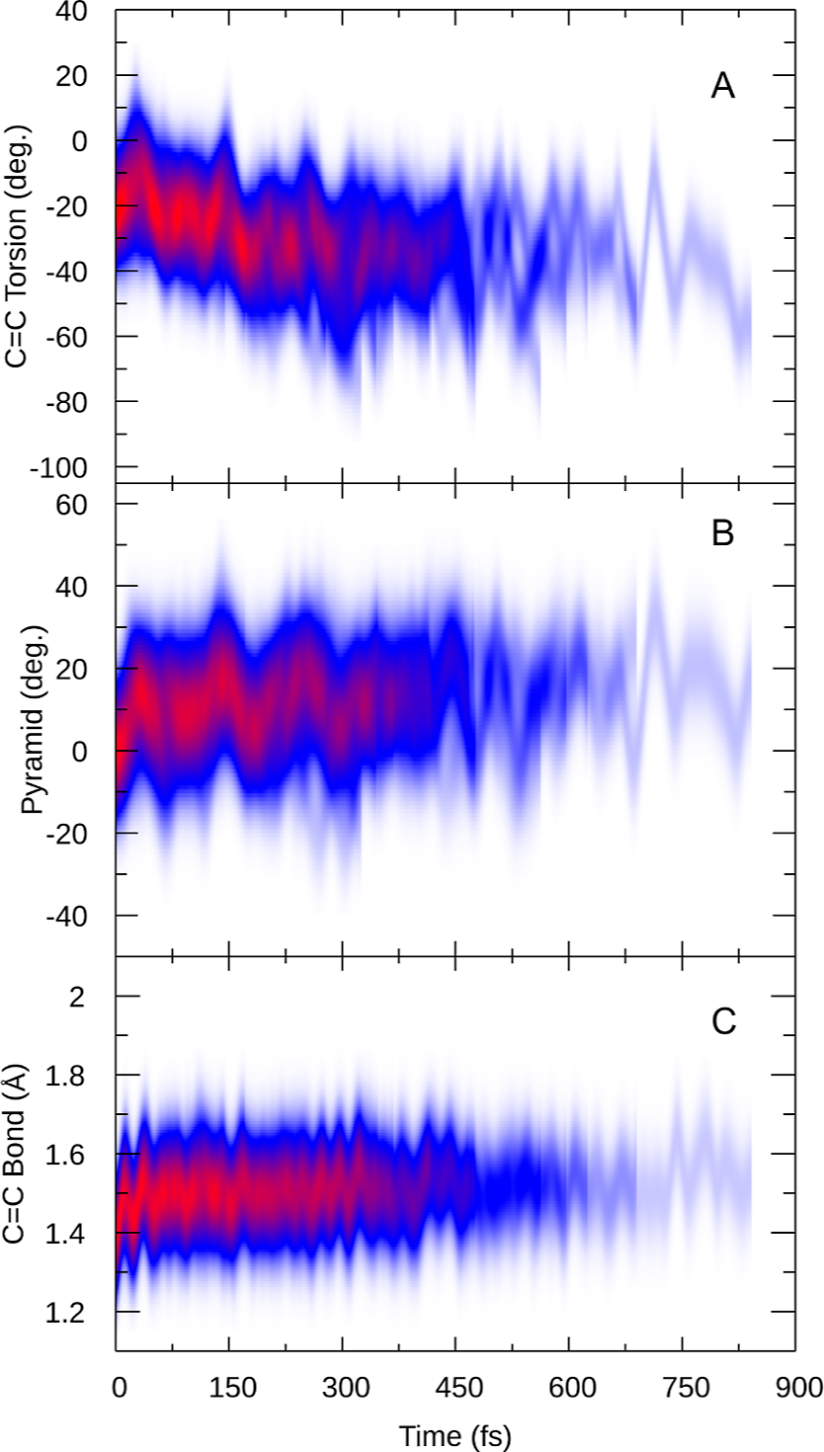
Time-resolved evolution of C=C torsion, pyramidalization, and bond length in the S_1_ state, convoluted with a 20° Gaussian in panels (a,b) and a 0.2 Å Gaussian in panel (c).

Figure 5 presents the time evolution of the most important geometry parameters after excitation until each respective trajectory approaches the S_1_/S_0_ crossing. In panel A, we plot the torsion angle of the double bond, which is one of the main reaction coordinates to reach the crossing region. We observe that, from the initial distribution around −20°, after excitation, the angle first momentarily increases (reaching angles of about 0° after 40 fs) for one oscillation. Subsequently, the torsion angle distribution slowly shifts to more negative values, reaching about −40° after about 300 fs. For longer times, we cannot follow the evolution as the trajectories reach the S_1_/S_0_ crossing, thus triggering the stopping criterion for our simulations. In Figure 5B, we plot the pyramidalization angle of the fluorene stator. It can be seen that the pyramidalization angle starts at 0° and subsequently increases quickly to about 10°, within only 40 fs, showing that the stator slightly bends in the excited state. This motion is on the same time scale as the temporary increase in the C=C torsion angle, which suggests that these two motions are coupled. The C=C bond length in Figure 5C also shows typical signs of excited double bond systems, with a stretching motion from 1.35 to 1.60 Å. This is due to the breaking of the π bond in the excited state that drives the isomerization reaction. As can be seen, during the time the trajectories remain in the excited state, the C=C bond length oscillates around 1.5 Å, indicating that the excited state maintains the wave function character.

**Figure 6:**
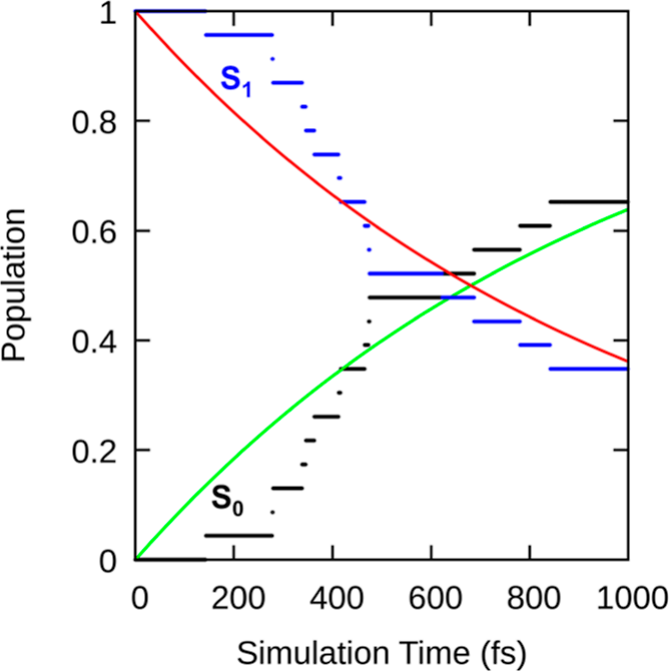
Time evolution of the S_1_ (blue) and S_0_ populations (black) and the exponential fit of the populations (red/green).

The evolution of the electronic populations of S_0_ and S_1_ is shown in Figure 6, computed under the assumption that the trajectories hop from S_1_ to S_0_ at the instances where the energy gap becomes smaller than 0.1 eV. As described elsewhere,^60^ this approach delivers a reasonable lower limit to the deactivation time scale of the excited state, even for methods like ADC(2) that do not describe the S_1_–S_0_ CI correctly. The plot shows that the population decreases nonexponentially. There is a short waiting time of about 170 fs before the first trajectory reaches the crossing region, and half of the trajectories have hopped to S_0_ after about 550 fs. A monoexponential fit delivers an effective overall decay time constant of τ_1_ = 982 fs. A similar nonexponential behavior was observed by other theoretical simulations on molecular motors.^27,29^ For the same system (but in the gas phase and using another electronic structure method), the authors found a very similar population decay with a waiting time of about 180 fs (taken where the S_1_ population is 0.95), a time of 550 fs until the decay of half of the population, and an effective exponential decay time constant of 750 fs. This agreement shows that indeed our energy gap-based approach can adequately capture the time scale of population decay. Note that we do not directly compare the electronic population decay of Figure 6 to experimental results because such comparison is more favorably done using simulated spectra, as presented in the next section.

### 3.3 Emission and Transient Absorption Spectra

Fluorescence up-conversion experiments have previously been employed to investigate the initial motion of this motor.^20^ In particular, the decay of signals in the emission spectra can be correlated to the lifetime of excited states. Based on the experimental spectrum, it has been suggested that a dark state with a low fluorescence transition moment is generated within 100 fs from a fast structural evolution of the bright Franck–Condon state along the S_1_ potential surface; then, the dark state undergoes slow radiationless decay to the ground state in about 1.5 ps.^20^ According to the emission signal, direct conversion from the S_1_ state to the ground state through a CI by fast internal conversion is unlikely.

**Figure 7:**
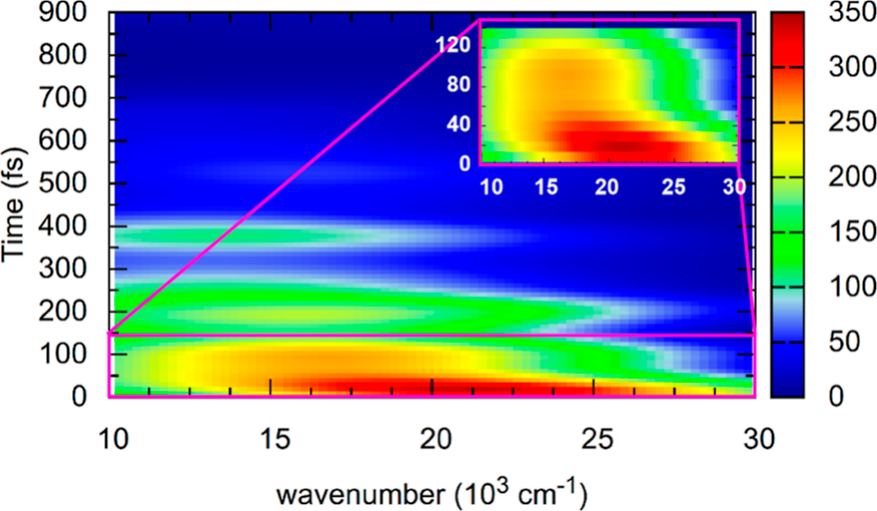
Simulated emission spectrum, convoluted by a 2.0 eV × 50 fs Gaussian, with a zoom of the first 150 fs shown in the inset.

To investigate this hypothesis, we simulate the time-resolved emission spectrum including solvent from our QM/MM-SHARC trajectories; see Figure 7. There is an initial intense emission centered at 22,000 cm^–1^, arising from the emission of the bright excited ππ* state. Within about 100 fs, the signal center shifts to 17,000 cm^–1^ (see the inset) and simultaneously loses a large fraction of its intensity. We assign this loss of intensity to the stretch and torsion of the C=C bond that reduces the relevant transition dipole moment, confirming the experimental interpretation. A red shift of ca. 4000 cm^–1^ is also observed in the experimental emission spectrum within 150 fs.^20^ There, a fast quenching with a large red shift was also predicted in a reverse *trans–cis* isomerization process. The redshift should occur in both *cis–trans* and *trans–cis* isomerizations, as has been confirmed by both theoretical predictions^27^ and experimental measurements.^20^ We note oscillations in the signal intensity and energy (between 15,000 and 16,000 cm^–1^) between 150 and 400 fs in Figure 7, which is consistent with the experimental time-resolved emission spectrum.^20^ The oscillations fit roughly with the oscillations observed in the pyramidalization angle (Figure 5B), but these might also be related to other vibrational degrees of freedom.

**Figure 8:**
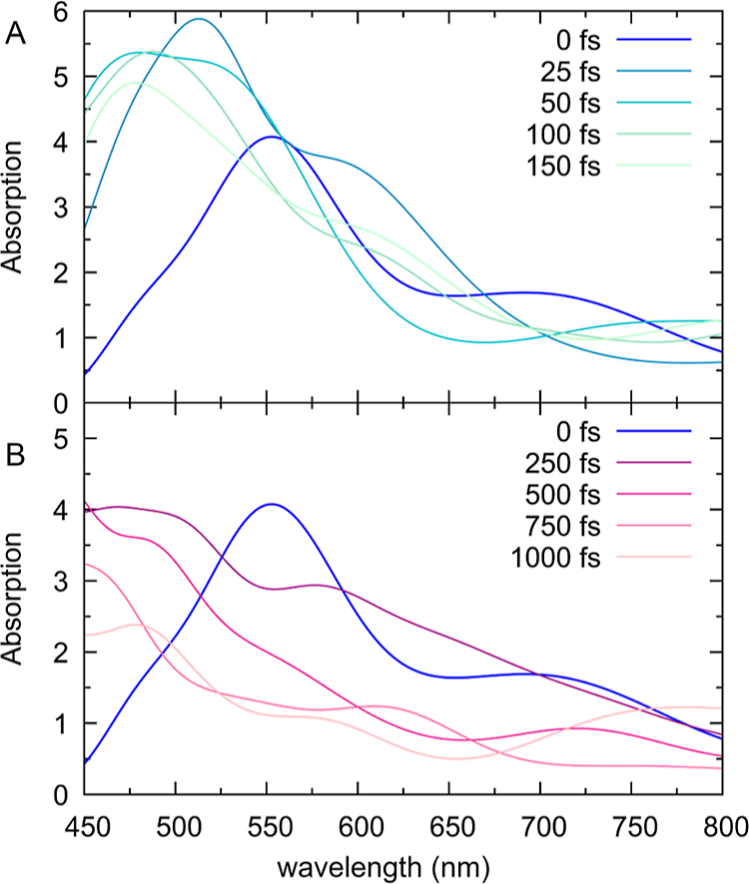
Simulated transient absorption spectra up to 1 ps: (a) 0–150 and (b) 250–1000 fs. Convolution was done with a 0.3 eV Gaussian, no convolution in the time domain was applied.

The excited-state dynamics can also be monitored with the help of transient absorption spectroscopy, where the S_1_ state is further excited to higher electronic states. Accordingly, we computed the transition energies and oscillator strengths from the active state of all trajectories to higher states up to about 3 eV above S_1_ in our QM/MM-SHARC trajectories. Figure 8 shows the simulated time-resolved absorption spectra of the *cis* configuration in DMSO.

**Figure 9:**
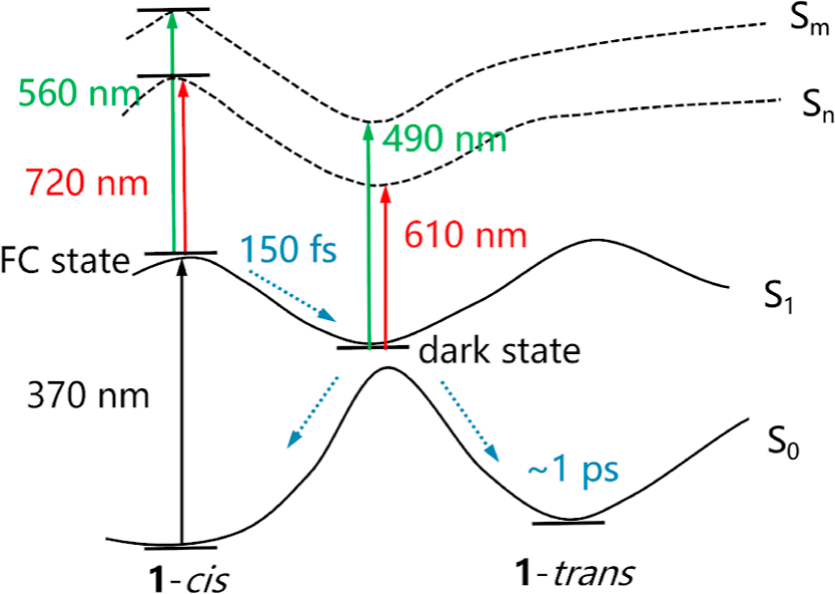
Schematic process of the kinetic steps during the formation of Franck–Condon (FC) and dark states along the S_1_ potential energy surface.

At 0 fs, our simulations predict a transient spectrum with a notable absorption band at 560 nm and a shoulder at around 720 nm. Within the first 150 fs, both features blue-shift to about 490 and 610 nm, respectively. After about 250 fs, both features start to decay (Figure 8B), although there is still substantial intensity left after 1 ps due to the trajectories that have not yet reached the S_1_–S_0_ crossing region. The simulated transient absorption spectrum is in reasonable agreement with previously reported experiments.^20,25^ The experimental spectrum in cyclohexane^25^ shows two bands at zero delay, centered at 760 and 550 nm. While the 760 nm band decays immediately and blueshifts slightly, the 550 nm signal grows for the first 120 fs before decaying over multiple ps. In the experimental work,^25^ the >700 nm signal was assigned to the bright Franck–Condon ππ* state, whereas the 550 nm absorption was assigned to a dark excited state that is populated within 150 fs. While our results mostly agree with this assignment, we want to point out that both the experiment and our simulations show considerable 550 nm absorption at *t* = 0. This indicates that the “dark” state might actually be the same electronic state as the bright state, although at different nuclear coordinates—and additionally indicates that the initial nuclear distribution is rather broad. Figure 9 summarizes these results for the initial dynamics of the molecular motor and the evolution of its transient absorption spectrum.

### 3.4 Solvation Effects

**Figure 10:**
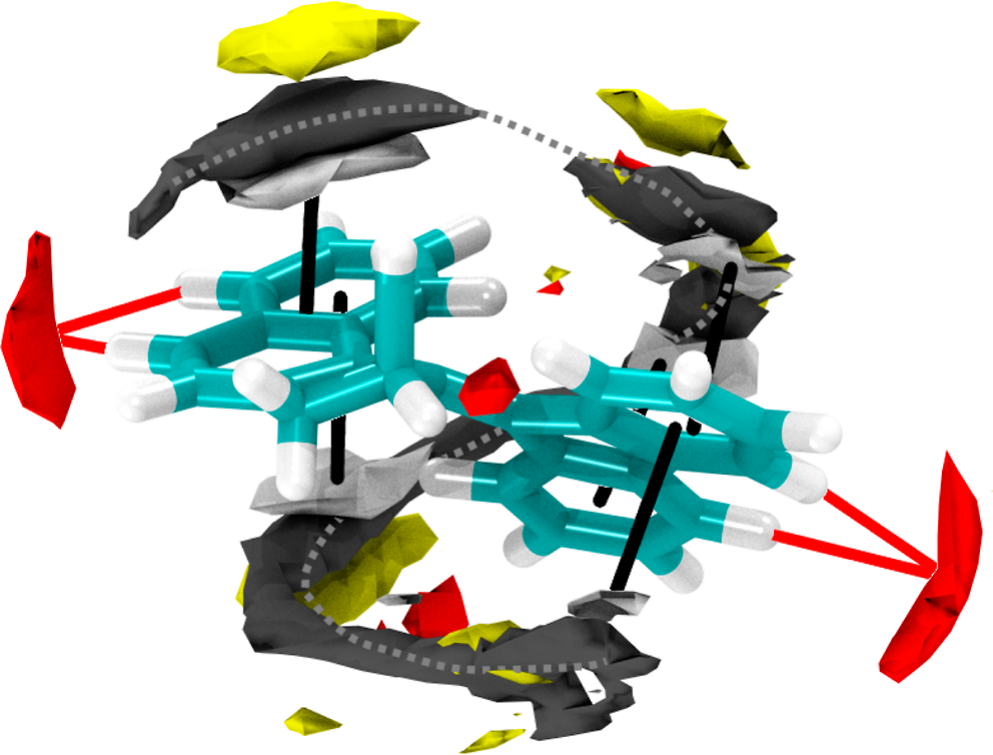
Three-dimensional distribution of solvent atoms (C gray, H white, S yellow, and O red) from classical MD simulations, showing significant solvent order around the molecule. Black lines indicate interactions of DMSO methyl groups with the π system of the rings. Red lines indicate interactions of the DMSO oxygen atoms with C–H groups. The statistical positions of the DMSO carbon atoms form a distinct spiral motif around the rings (gray dotted line). Isovalues are chosen to be just above the noise level: 3.9 for C and S, 2.1 for H, and 4.9 for O (e.g., red volumes indicate regions where O is 4.9 times as likely found as in bulk solution).

We first analyze the solvation structure predicted by the 10 ns classical MD trajectory of the electronic ground state in DMSO. Figure 10 shows the three-dimensional distribution of the solvent atoms (obtained as a grid-based histogram with 0.5 Å resolution). With the force field used, the molecule exhibits a distinct and very nonuniform first solvation shell with two different types of interactions. The π systems of the aromatic rings show interactions with the methyl groups of several DMSO molecules. Here, the naphthalene ring is coordinated by two DMSO molecules (one on each side) and the fluorene ring by three (two in the back of Figure 10, one in the front). Additionally, the C–H bonds of the molecule form weak, hydrogen-bond-like interactions with oxygen atoms of the DMSO molecules. Of particular notice is that the DMSO solvent forms a distinct spiral motif around the molecule, presumably leading to a relatively rigid solvent cage that can hamper the isomerization of the rotor upon excitation.

**Figure 11:**
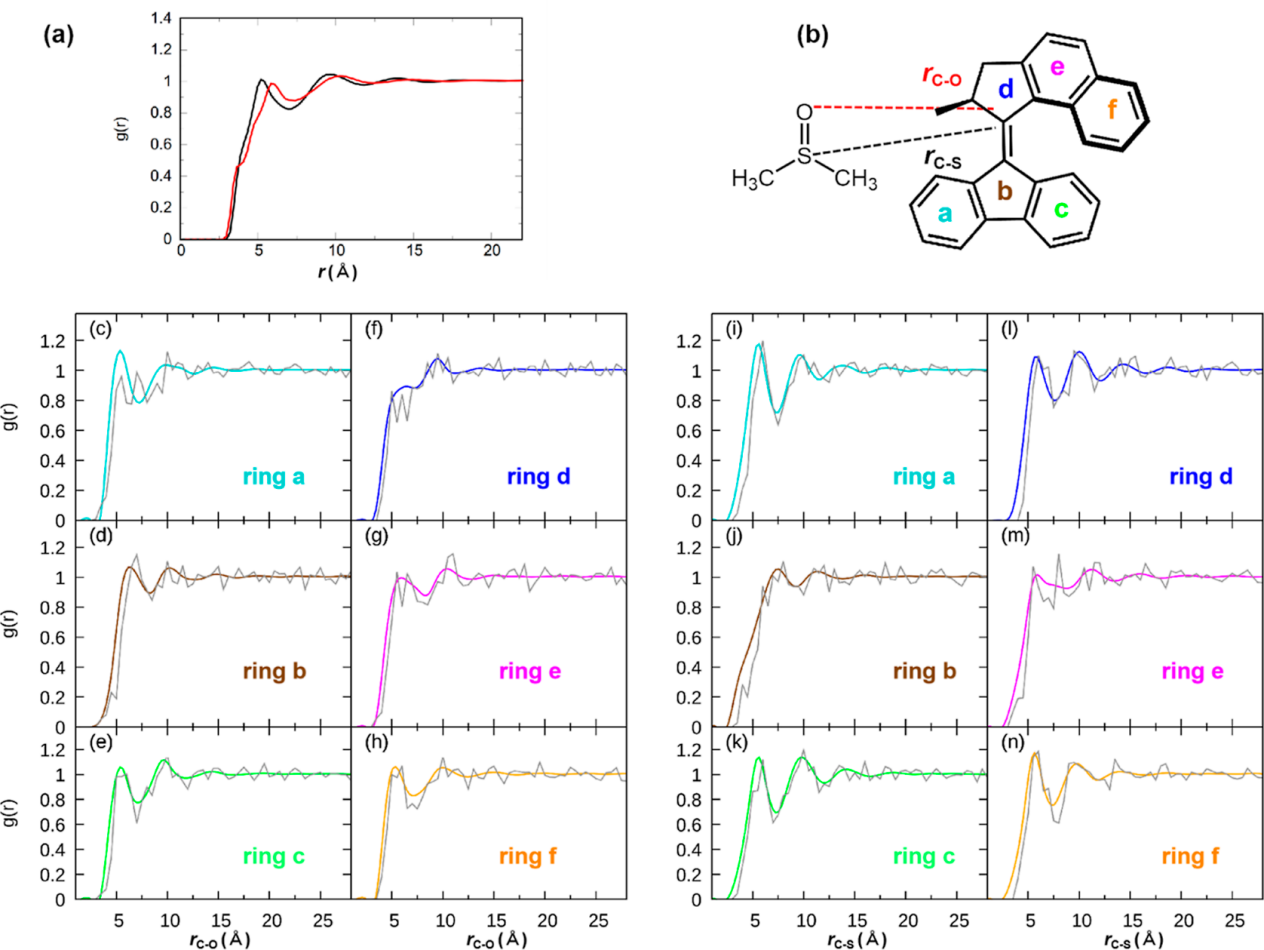
(a) RDFs *g*(*r*) between all motor carbons and the DMSO solvent (red for *r*_C–O_ and black for *r*_C–S_) with the distance defined in (b). (c–h) *g*_C–O_(*r*) for ground (color) and excited states (gray). (i–n) *g*_C–S_(*r*) for ground (color) and excited states (gray). Different colors correspond to the six rings, as indicated in (b). RDFs in the ground state are from classical MD, and excited-state RDFs were derived from SHARC simulations by averaging over the last 100 fs of each trajectory.

There is not a sufficient number of excited-state trajectories to perform a similar three-dimensional analysis in the electronic excited state. Instead, we calculate RDFs, even though they do not display details about the three-dimensional spatial arrangement of the solvent, as they require much less trajectory data to reach a reasonable signal-to-noise level. The distance distributions between all carbon atoms of **1**-*cis* and oxygen/sulfur atoms in DMSO, noted as *r*_C–O_/*r*_C–S_, respectively, are analyzed in Figure 11a. These RDFs exhibit only little structure, with the first weak peak at 5 Å and the second one around 9–10 Å. A small shoulder in the C–O RDF appears at 4 Å, presumably arising from the C–H···O interactions mentioned above. We also note that the first peak of the *r*_C–S_ RDF is closer than the first peak of the *r*_C–O_ RDF, indicating that the motor is more often coordinated by the methyl groups of DMSO compared to the oxygen atom.

The RDFs in Figure 11a do not exhibit much structure due to the averaging over all of the motor carbon atoms. In order to provide a larger degree of detail in the RDFs, we plot separate RDFs for each of the six rings (see ring labeling in Figure 11b) in Figure 11c–h [for *g*_C–O_(*r*)] and Figure 11i–n [for *g*_C–S_(*r*)]. The colored lines in the plots show the ground-state RDFs (where each color corresponds to one of the rings), and the gray lines show the excited-state RDFs, which we will discuss further below.

**Figure 12:**
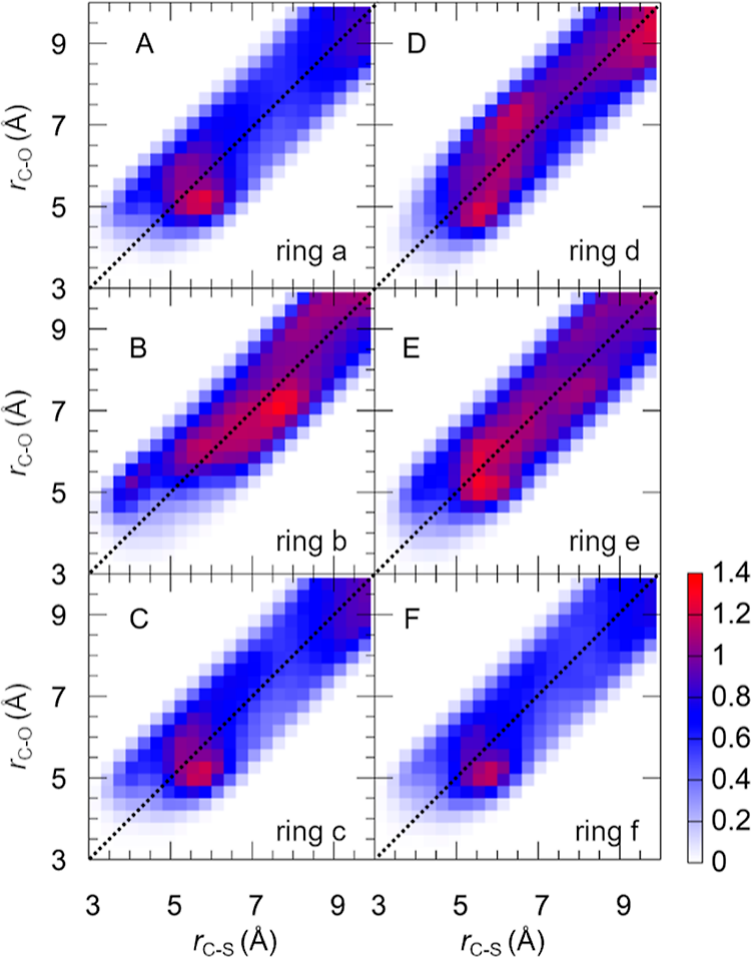
(A–F) Correlations of RDFs [*g*_C–S_(*r*) and *g*_C–O_(*r*)] as projections onto a plane composed of distances between DMSO and a–f rings of **1**-*cis* in the ground state (see Figure 11b for the ring labels). Note that the width of the distributions is determined by the DMSO C=S bond length.

The rings a, c, and f in Figure 11 constitute the terminal rings of the motor, providing an easier approach to DMSO. For these rings, the *g*_C–O_(*r*) RDFs (panels c, e, and h) are very similar and show a first maximum at about 5.5 Å and a first minimum at 7.5 Å. These peaks show maximum values slightly above 1, indicating weak but favorable interactions with the DMSO oxygen atoms. The three rings also show very similar *g*_C–S_(*r*) RDFs (panels i, k, and n), which also have a peak near 5.5 Å and a minimum near 7.5 Å. One explanation for the similarity of the C–S and C–O RDFs could be that DMSO arranges predominantly with the S=O bond parallel to the rings. However, as shown in Figure 10, some DMSO molecules arrange with the methyl groups toward the rings, so we assume that the similarity of the C–S and C–O RDFs is rather due to averaging over differently arranged DMSO molecules. The 2D correlation RDFs in Figure 12 provide additional insight into the DMSO orientation. There, in panels A, C, and F a slight peak can be seen at the coordinates 6 Å (C–S), 5 Å (C–O), indicating that oxygen is more often closer to the rings than sulfur. However, the correlation RDFs also show that DMSO can be found at coordinates 5–7 Å (C–S) and 5–7 Å (C–O), which shows that the orientational preference of DMSO is rather weak.

The rings b, d, and e in Figure 11 are more sterically hindered (compared to the terminal a, c, and f rings) and thus cannot be as easily approached by solvent. According to the RDFs, ring b (panels d and j) is the most screened ring, given that its C–O RDF peaks only at 6.4 Å and its C–S RDF at 7.2 Å. For rings d and e, the RDFs (panels f, g, l, and m) exhibit peaks and minima at approximately the same positions as for the terminal rings; however, a smaller density is observed, indicating less structure in the first solvation shell. These findings are supported by the correlation RDFs in Figure 12—compared to clear peaks at short distances for rings a, c, and f, the rings b, d, and e show similar values across the entire distribution. Interestingly, the correlation RDFs also reveal slightly different solvation structures for the b, d, and e rings—in particular, a weak peak at coordinates 4 Å (C–S), 5.5 Å (C–O).

Besides the ground-state RDFs in Figure 11 (black lines), we also show the excited-state RDFs (gray lines) obtained from the last 100 fs of the QM/MM-SHARC simulations. Due to the small number of trajectories, the RDFs in the S_1_ state are quite noisy. However, within statistical limits, the excited-state RDFs match with the ground-state ones, showing that the solvent shell probably does not strongly rearrange due to the excitation. It can also be seen that the left edge of all the C–O and C–S RDFs shifts slightly to larger values, hinting at a fast response of the solvent molecules to the excitation,^61,62^ possibly due to the initiated motion of the motor that pushes away and disrupts the solvation shell.

**Figure 13:**
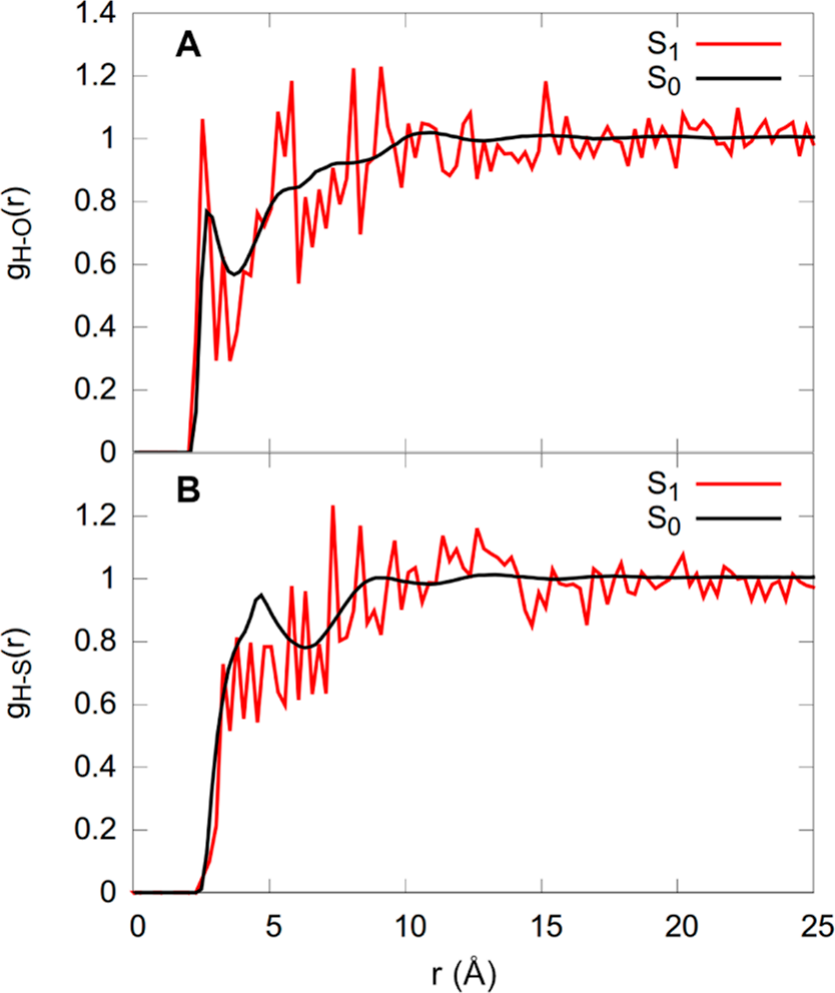
RDFs of **1**-*cis* (A) *g*_H–O_(*r*) and (B) *g*_H–S_(*r*) in the electronic ground (black) and excited (red) states.

Lastly, we briefly discuss the possible formation of C–H···O and C–H···S weak hydrogen bonds between the motor and DMSO. The relevant RDFs are shown in Figure 13, with the ground state in black and the excited state in red. The ground state RDFs show that overall the solvation of the motor is not dominated by such hydrogen bonds as no peaks exceed a value of 1. However, there is a notable narrow peak in the H–O RDF at about 2.9 Å—in agreement with the C–H···O interactions seen in Figure 10—showing that hydrogen bonding plays a role for some sections of the motor solvation shell. The feasibility of such C–H···O interactions is supported by an optimized hydrogen-bonded motor-DMSO complex at the B3LYP-D3/cc-pVDZ level of theory as shown in Figure S1a. This optimization provides a binding energy of 13 kcal/mol with an O···H distance of 2.2 Å and a C–H···O angle of 151.2°. Conversely, given the low values of the H–S RDFs and the location of the first peak (4.7 Å), it does not appear that C–H···S hydrogen bonds are formed. An attempt to optimize a C–H···S-bonded motor-DMSO complex produced a structure that shows minimal interaction of C–H with S (distance of 3.6 Å and a C–H···S angle of 77.9°) and instead shows noncovalent bonding through the methyl groups (Figure S1b).

### 3.5 Conclusions

We computed the excited-state dynamics of a second-generation overcrowded alkene molecular motor solvated in DMSO using ab initio MD simulations. Whereas the motor is rather rigid in the ground state, after excitation to the bright S_1_ state, we observe simultaneous torsion, pyramidalization, and stretching of the C=C moiety. This nuclear motion brings the wave packet toward the S_1_–S_0_ crossing region, which the first trajectories reached after about 170 fs. After 550 fs, half of the trajectories have formally decayed to the ground state, and an overall exponential decay time constant of 980 fs was found.

The nuclear and electronic dynamics can be observed in the time-resolved emission spectrum, which shows a significant redshift within the first 150 fs due to nuclear motion away from the Franck–Condon region. The initial reduction in emission intensity is coupled to the torsion, pyramidalization, and stretching of the C=C bond, all of which reduce the S_1_–S_0_ transition dipole moment. At later times, the emission signal decays due to relaxation to the electronic ground state with a time constant of about 1 ps. Experimentally observed oscillations in the emission spectrum were reproduced and assigned to the pyramidalization motion. The simulated transient absorption spectra reproduce the experimental absorption bands at about 550 and 700 nm. We assign these bands to two different higher-lying electronic states, whose transition from the S_1_ is bright. The excited-state dynamics led to shifts and an increase in the 550 nm band within the first 150 fs, followed by a decay of the signal due to the excited-state decay.

We also, for the first time, report the structure of the first solvation shell of the investigated motor in DMSO. The solute–solvent dynamics is dominated by the interaction of DMSO methyl groups with the ring π systems on one hand and the interaction of DMSO oxygen atoms with the ring C–H groups on the other hand. Several DMSO molecules form a rigid spiral motif around the motor in the ground state, explaining partially the viscosity dependence of the isomerization dynamics. In the excited state, we observe a slight shift of all RDFs to larger values, consistent with an inertial solvent response, where the solvent recedes from the molecule due to changes in the solute electron density. We do not observe any other significant changes in the solute–solvent RDFs, which we attribute to the rather small change in polarity of the molecule due to the delocalization of the excited electrons. In summary, our work suggests that the ground state solvent distribution might have a greater impact than the solvent response in the excited state for this class of molecular motors and that molecular modifications that disrupt the formation of a rigid solvent cage might improve the performance of the motor.

## Supporting Information Available

Optimized coordinates of **1**-*cis* in the ground state and optimized coordinates and graphical depiction of two hydrogen-bonded complexes with DMSO (PDF)

## Notes

Published as part of *The Journal of Physical Chemistry A*virtual special issue “Women Scientists in China”.
