# Supplementary material for: Excited-State Dynamics Simulations of a Light-Driven Molecular Motor in Solution
Source: J Phys Chem A. 2023 Nov 2;127(45):9520–9. doi: 10.1021/acs.jpca.3c05841 (PMC10658450; doi:10.1021/acs.jpca.3c05841)
Supplement: Supplementary file 1 — jp3c05841_si_001.pdf [file jp3c05841_si_001.pdf]

Supporting information: Excited-State Dynamics Simulations of a Light-Driven Molecular Motor in Solution

Jin Wen,*,†,‡ Sebastian Mai,‡ and Leticia González‡

†*State Key Laboratory for Modification of Chemical Fibers and Polymer Materials, College of Materials Science and Engineering, Donghua University, Shanghai 201620, China*

‡*Institute of Theoretical Chemistry, Faculty of Chemistry, University of Vienna, Währinger Str. 17, 1090 Vienna, Austria*

E-mail: jinwen@dhu.edu.cn

Contents

Coordinates of 1-*cis* in S_0 state **S3**

Coordinates of 1-*cis*–DMSO noncovalent complexes **S4**

Depiction of 1-*cis*–DMSO noncovalent complexes **S6**

Coordinates of 1-*cis* in S_0 state

Table S1: Coordinates of optimized 1-*cis* minimum in the S_0 state at B3LYP-D3/cc-pVDZ level.

C	1.783092	3.776495	-1.103234
C	2.762550	2.894788	-0.637314
C	2.401188	1.581649	-0.328399
C	1.058802	1.135814	-0.453837
C	0.098914	2.019855	-0.965543
C	0.465828	3.333208	-1.279583
C	3.230641	0.447301	0.080090
C	2.404879	-0.706015	0.168562
C	0.997592	-0.315612	-0.127453
C	2.986798	-1.918826	0.566211
C	4.597409	0.387410	0.358618
C	5.163142	-0.834786	0.733152
C	4.359052	-1.976484	0.837105
H	4.803967	-2.927357	1.139009
H	2.395291	-2.824054	0.682281
H	5.217441	1.284141	0.288755
H	6.231382	-0.898063	0.952001
H	3.799040	3.224542	-0.535082
H	2.049217	4.806940	-1.349446
H	-0.288946	4.018726	-1.671460
H	-0.926791	1.700553	-1.134891
C	-0.090795	-1.151000	-0.162788
C	-0.009900	-2.669057	-0.362362
C	-1.531426	-0.831321	-0.254682
C	-1.175006	-2.912436	-1.366020
C	-2.162308	-1.833933	-0.995266
C	-0.233847	-3.468116	0.934643
H	0.951187	-2.952333	-0.810780
C	-2.317665	0.183476	0.387728
C	-3.542563	-1.787934	-1.277717
C	-4.302099	-0.757849	-0.760129
C	-3.727064	0.221221	0.099727
H	-4.004919	-2.565828	-1.890247
H	-5.373654	-0.703676	-0.968929
H	-1.603031	-3.924653	-1.289003
H	-0.817028	-2.783088	-2.402812
H	-1.229056	-3.256580	1.357652
H	0.512139	-3.217810	1.704137
H	-0.170951	-4.550853	0.736123
C	-4.534211	1.216036	0.720108
C	-3.993865	2.109810	1.619711
C	-2.617497	2.036461	1.945884
C	-1.800760	1.100440	1.343839
H	-5.600833	1.245803	0.482682
H	-4.626372	2.863209	2.094412
H	-2.198401	2.725532	2.682497
H	-0.744957	1.051281	1.606785

Coordinates of 1-*cis*–DMSO noncovalent complexes

Table S2: Coordinates of the optimized structure of S=O$\cdots$H bonded motor-DMSO complex at the B3LYP-D3/cc-pVDZ level.

C	1.93049500	3.93698200	-0.61281900
C	3.12635300	3.30559900	-0.24515200
C	3.10821700	2.01528100	0.29558600
H	4.03458200	1.49610600	0.55179300
H	4.07926300	3.82120800	-0.38590100
H	1.95622400	4.94280400	-1.03870600
C	0.69665100	3.29563500	-0.44978900
H	-0.20614000	3.81092200	-0.76873800
C	0.65264200	2.00885900	0.10662400
C	1.87521600	1.38085000	0.46826300
C	1.57235500	0.05140600	1.00232200
C	2.42374300	-0.90113100	1.57062300
H	3.50252600	-0.73036000	1.58754300
C	1.86183800	-2.06938900	2.09813900
H	2.50897500	-2.82668400	2.54805900
C	0.47257900	-2.25882200	2.08364800
H	0.04168300	-3.15894000	2.52811200
C	-0.37758500	-1.30545400	1.51227300
H	-1.45279000	-1.46647000	1.53645800
C	0.16942800	-0.15326400	0.93125500
C	-0.46558600	1.05430800	0.33927900
C	-1.79644500	1.28119500	0.10609300
C	-2.44017700	2.66670800	0.01169400
C	-2.73920000	3.08402900	-1.43909800
H	-3.20583200	4.08231300	-1.46718900
H	-3.43165300	2.36750700	-1.90992600
H	-1.82413400	3.11238900	-2.04962500
H	-1.81271700	3.42467600	0.49827700
C	-3.76155800	2.45208100	0.80676700
H	-4.57308900	3.12158800	0.48029200
H	-3.59198900	2.63413600	1.88252700
C	-4.06970300	0.99833600	0.54943500
C	-5.30630000	0.33577700	0.67924500
C	-5.40319600	-0.98959400	0.30292500
H	-6.35222600	-1.52285500	0.40155300
H	-6.17732000	0.87024700	1.06584200
C	-2.92707700	0.33385500	0.09985700
C	-3.03069700	-1.00175600	-0.40977200
C	-4.29420100	-1.67503400	-0.27111200
C	-4.42283100	-3.00788000	-0.75385200
H	-5.38105800	-3.51911100	-0.62976500
C	-3.37208600	-3.63857900	-1.38545600
H	-3.48628500	-4.65979900	-1.75573800
C	-2.14727200	-2.95189300	-1.57541800
H	-1.32811500	-3.44333700	-2.10512900
C	-1.98096800	-1.66739100	-1.09945000
H	-1.03744600	-1.14538300	-1.25095100
S	5.50899600	-1.06728100	-0.78558800
C	4.01957200	-0.61903500	-1.77288400
O	5.44579700	-0.28602500	0.54073700
C	4.97823000	-2.78725700	-0.39368600
H	3.11701800	-0.77723000	-1.16600100
H	4.00631600	-1.23332300	-2.68639100
H	4.11463900	0.44521100	-2.02689600
H	4.96271600	-3.38559700	-1.31763200
H	3.98671900	-2.75665500	0.08128100
H	5.72520700	-3.18520200	0.30711100

Table S3: Coordinates of the optimized structure of O=S$\cdots$H bonded motor-DMSO complex at the B3LYP-D3/cc-pVDZ level.

C	-2.97519300	2.40004600	2.14849600
C	-3.80498600	1.30037500	2.40613100
C	-3.39263800	0.01331200	2.04987600
H	-4.03238900	-0.84940100	2.24447900
H	-4.77419100	1.44977700	2.88655300
H	-3.30100000	3.40282000	2.43342600
C	-1.72891400	2.23548500	1.53167200
H	-1.10772300	3.11244200	1.36747200
C	-1.30595900	0.95247800	1.15149600
C	-2.15241600	-0.15468800	1.43103700
C	-1.50579700	-1.36945600	0.93797000
C	-2.00078000	-2.67187800	0.84839500
H	-2.98515700	-2.91829100	1.25060700
C	-1.23385100	-3.64291300	0.19860200
H	-1.60656200	-4.66600700	0.11533400
C	-0.00147400	-3.29823700	-0.37523800
H	0.57846500	-4.05528700	-0.90761000
C	0.49599900	-1.99369300	-0.28774000
H	1.43843900	-1.74907000	-0.77142500
C	-0.24067700	-1.01889100	0.39905600
C	-0.05084300	0.45013900	0.53034700
C	1.00393500	1.20815800	0.09175800
C	0.92217700	2.69648900	-0.25383500
C	1.51518200	3.59328900	0.84739600
H	1.44211100	4.65546500	0.56258600
H	2.57842000	3.35209400	1.00721100
H	0.99585000	3.45544200	1.80772300
H	-0.11357700	2.99505000	-0.46273700
C	1.78674500	2.76559100	-1.54669300
H	2.25875400	3.74947200	-1.69567700
H	1.16237100	2.55873600	-2.43366700
C	2.79418300	1.66474000	-1.33031100
C	4.04123700	1.49215600	-1.96255700
C	4.86261800	0.45823900	-1.55775500
H	5.83148800	0.30392400	-2.03958400
H	4.35370100	2.17419500	-2.75670300
C	2.34749800	0.78560100	-0.34154900
C	3.22836300	-0.21997100	0.17624500
C	4.49839900	-0.39413700	-0.47588300
C	5.38949900	-1.39478500	0.00389400
H	6.34619800	-1.53217300	-0.50661900
C	5.06768500	-2.16334900	1.10239400
H	5.76256600	-2.92475500	1.46323200
C	3.84161600	-1.95001100	1.77947800
H	3.60434800	-2.53886700	2.66813600
C	2.94498600	-1.00291900	1.32792500
H	2.00622600	-0.84453400	1.85650800
S	-4.08367400	-0.66598600	-1.37279300
C	-4.35041800	1.15487400	-1.24540200
O	-5.07645800	-1.17383400	-2.42277900
C	-2.45416400	-0.54980000	-2.23076900
H	-4.32976400	1.57004500	-2.26449800
H	-3.58483100	1.60617300	-0.59754000
H	-5.34836500	1.29094700	-0.80618400
H	-1.75991800	0.08869000	-1.66722400
H	-2.66441000	-0.14793500	-3.23336600
H	-2.05266200	-1.56997400	-2.30013300

Depiction of 1-*cis*–DMSO noncovalent complexes

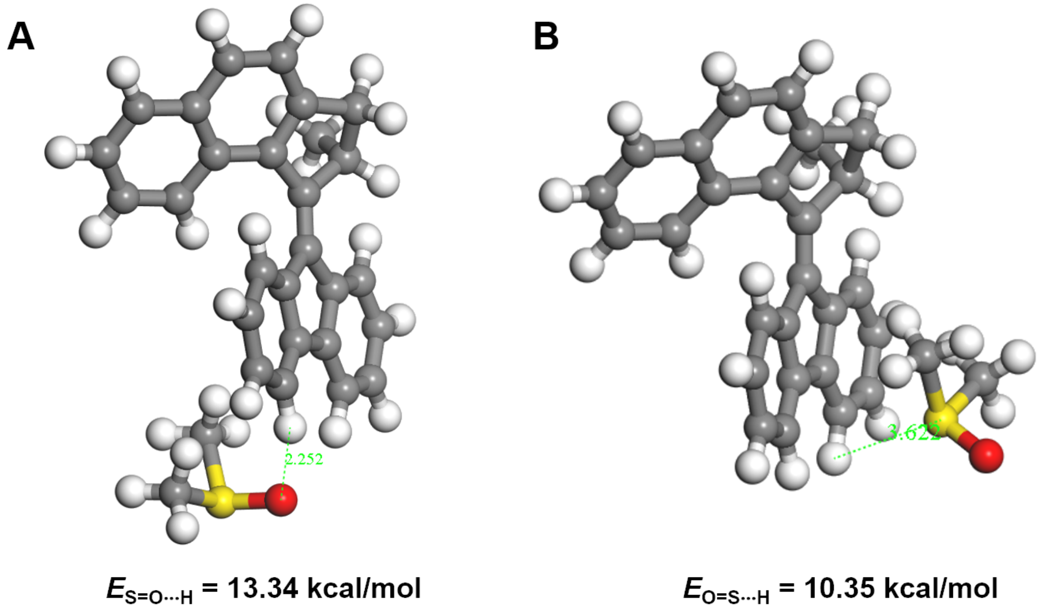

Figure S1: Optimized structures of a molecular motor with a DMSO molecule, where weak hydrogen bonds between S=O bonds of DMSO and hydrogen atoms of motors are formed as (A) S=O$\cdots$H and (B) O=S$\cdots$H bonds. The distances between O/S atoms to hydrogen atoms are labeled as $R_{\text{O-H}}$ = 2.252 Å and $R_{\text{S-H}}$ = 3.622 Å respectively.
